# Impact of corticosteroid doses on prognosis of severe and critical COVID-19 patients with Omicron variant infection: a propensity score matching study

**DOI:** 10.1007/s10787-024-01520-0

**Published:** 2024-08-09

**Authors:** Shiyao Wang, Ziying Chen, Xinran Zhang, Xiaojing Wu, Yuqiong Wang, Qi Zhang, Linna Huang, Xiaoyang Cui, Ying Cai, Xu Huang, Jingen Xia, Sichao Gu, Min Li, Qingyuan Zhan

**Affiliations:** 1 National Center for Respiratory Medicine; State Key Laboratory of Respiratory Health and Multimorbidity; National Clinical Research Center for Respiratory Diseases; Institute of Respiratory Medicine, Chinese Academy of Medical Sciences; Department of Pulmonary and Critical Care Medicine, Center of Respiratory Medicine, China-Japan Friendship Hospital, #2 Yinghuayuan East Street, Chaoyang District, Beijing, 100029 China; 2 National Center for Respiratory Medicine; State Key Laboratory of Respiratory Health and Multimorbidity; National Clinical Research Center for Respiratory Diseases; Institute of Respiratory Medicine, Chinese Academy of Medical Sciences; Department of Clinical research and Data management, Center of Respiratory Medicine, China-Japan Friendship Hospital, Beijing, 100029 China

**Keywords:** COVID-19, Omicron variant, Critically ill, Prognosis, Corticosteroid

## Abstract

**Background:**

There is lack of research on corticosteroid use for severe and critical COVID-19 patients with Omicron variant infection.

**Methods:**

This multi-center retrospective cohort study involved 1167 patients from 59 ICUs across the mainland of China diagnosed with severe or critical SARS-CoV-2 Omicron variant infection between November 1, 2022, and February 11, 2023. Patients were segregated into two groups based on their corticosteroid treatment—usual dose (equivalent prednisone dose 30–50 mg/day) and higher dose (equivalent prednisone dose > 50 mg/day). The primary outcome was 28-day ICU mortality. Propensity score matching was used to compare outcomes between cohorts.

**Results:**

After propensity score matching, 520 patients in the usual dose corticosteroid group and 260 patients in the higher dose corticosteroid group were included in the analysis, respectively. The mortality was significantly higher in the higher dose corticosteroid group (67.3%, 175/260) compared to the usual dose group (56.0%, 291/520). Logistic regression showed that higher doses of corticosteroids were significantly associated with increased mortality at 28-day (OR = 1.62,95% CI 1.19–2.21, *p* = 0.002) and mortality in ICU stay (OR = 1.66,95% CI 1.21–2.28, *p* = 0.002). Different types of corticosteroids did not affect the effect.

**Conclusions:**

The study suggests that higher-dose corticosteroids may lead to a poorer prognosis for severe and critical COVID-19 patients with Omicron variant infection in the ICU. Further research is needed to determine the appropriate corticosteroid dosage for these patients.

## Introduction

Corticosteroids are used to treat severe COVID-19 due to their effectiveness in managing inflammation (Lamers & Haagmans 2022; Wu et al. 2020). They are considered essential in the treatment of critically ill patients (Angus et al. 2020; Horby et al. 2021; Tomazini et al. 2020). However, the appropriate dosage for severe or critical cases remains a topic of debate. Studies have shown benefits with doses like dexamethasone 6 mg daily or hydrocortisone 200 mg daily (Angus et al. 2020; Horby et al. 2021). Some studies suggested higher doses such as dexamethasone 10–20 mg per day and methylprednisolone 80 mg per day are also effective (Tomazini et al. 2020; Wu et al. 2020). The question of whether higher doses are more beneficial requires further investigation, as previous studies have provided inconsistent results (Granholm et al. 2022; Group 2023; Munch et al. 2021; Salvarani et al. 2022).

Additionally, the majority of patients in previous studies were infected with SARS-CoV-2 non-Omicron variants, while the Omicron variant has become dominant since 2022. Since severe or critical cases are less common with Omicron variant infection, there is a lack of research on the use of corticosteroids for this specific group. It is important to explore whether higher doses of corticosteroids are necessary for critically ill patients with Omicron variant infections, considering the potential lower inflammatory response.

Therefore, this study aims to explore whether the application of higher doses of corticosteroids (equivalent prednisone doses of > 50 mg per day for the application of dexamethasone, methylprednisolone, prednisone, or prednisolone) compared to the application of usual doses of corticosteroids (equivalent prednisone doses of 30–50 mg per day) conducted a prognostic impact on severe or critical COVID-19 patients with Omicron variant infection through a multi-center retrospective cohort in the mainland of China.

## Materials and methods

### Study design and data collection

This is a multi-center retrospective cohort study including 59 ICUs in the mainland of China. Inclusion criteria were age > 18 years old, confirmed SARS-CoV-2 infection (COVID-19), and classified as severe or critical, requiring ICU care between November 1, 2022, and February 11, 2023. Exclusion criteria included a lack of prognostic information and unclear respiratory support modalities.

All demographic, clinical characteristics, laboratory tests, treatment, and outcome data were collected through electronic medical records and organized in standard data collection forms. All investigators were properly trained before data entry, and all data were reviewed by two supervisors. Because of the observational nature of the study, written informed consent was waived. The study protocol was approved by the Research Ethics Commission of China-Japan Friendship Hospital (2019-79-K51-1).

### Diagnostic criteria and definitions

The severity of the disease was based on the Chinese management guideline for COVID-19 (Trial Version 10.0), (China 2023) which included mild, moderate, severe, and critical. The diagnosis of COVID-19 was confirmed by polymerase chain reaction (PCR) or antigen testing using nasopharyngeal swabs or lower respiratory tract specimens (the sputum, transtracheal aspirates, bronchoalveolar lavage fluid). Severe or critical COVID-19 was defined if met any of the following criteria: (1) Shortness of breath with respiratory rates over 30 per minute; (2) Pulse oxygen saturation (SpO_2_) ≤ 93% in the resting state; (3) Partial pressure of oxygen/Fraction of inspired oxygen (PaO_2_/FiO_2_) ≤ 300 mmHg; (4) Respiratory failure requiring invasive mechanical ventilation; (5) Shock; (6) Combined with other organ dysfunction requiring ICU monitoring and treatment.

Immunosuppressed hosts include patients with primary immunodeficiency, active malignancy receiving treatment, AIDS, solid organ or bone marrow stem cell transplantation, long-term glucocorticoid, and immunosuppressive therapy for primary disease. Respiratory support, vasopressor use, PaO_2_, PaO_2_/FiO_2_ ratio (PFR), acute physiology and chronic health evaluation II (APACHE II), and sequential organ failure assessment score (SOFA) scores were the worst conditions or values for the first 24 h of ICU admission. Simple oxygen therapy refers to nasal cannula oxygen support or mask oxygen inhalation. Antiviral therapy prior to ICU admission and in combination includes Paxlovid, azvudine, and molnupiravir.

### Outcomes

The primary clinical outcome of the study was 28-day mortality in ICU stay. The secondary clinical outcome was in-ICU mortality. The other admission data included organ dysfunction in ICU stay, secondary infection, septic shock, acute kidney injury, acute myocardial injury, deep venous thrombosis, acute pulmonary embolism, acute liver injury, gastrointestinal bleeding, hyperglycemia, renal replacement therapy in ICU stay, extracorporeal membrane oxygenation (ECMO) in ICU stay.

Secondary infection refers to any infection that occurs beyond 48 h after ICU admission from the blood, respiratory tract, urinary system, or other sterile sites. Septic shock was diagnosed according to the definition provided by the Surviving Sepsis Campaign Guidelines for Management of Sepsis and Septic Shock: 2016 (Rhodes et al. 2017). Acute kidney injury was diagnosed based on the Kidney Disease: Improving Global Outcomes (KDIGO) 2012 clinical practice guidelines (Kellum & Lameire 2013). Serum cardiac biomarkers above 99th percentile upper reference limits or an abnormal electrocardiogram or echocardiography were indicators of acute myocardial injury (DeFilippis et al. 2019). Acute liver injury was defined as total bilirubin levels > 171 μmol/L or daily elevations ≥ 17.1 μmol/L and international normalized ratio (INR) ≥ 2.0 (Shen et al. 2019). Hyperglycemia was defined as random blood glucose > 250 mg/dl.

### Main exposure

Corticosteroid treatment refers to the administration of corticosteroids (any of methylprednisolone, prednisone, prednisolone, dexamethasone, or hydrocortisone) via oral or intravenous and at least one dose in ICU. The usual dose of corticosteroids was defined as an equivalent prednisone dose of 30–50 mg per day (hydrocortisone 120–200 mg per day, or prednisone/prednisolone 30–50 mg per day, or methylprednisolone 24–40 mg per day, or dexamethasone 4.5–7.5 mg per day). In comparison, the higher dose of corticosteroids was defined as an equivalent prednisone dose higher than 50 mg per day (hydrocortisone > 200 mg per day, or prednisone/prednisolone > 50 mg per day, or methylprednisolone > 40 mg per day, or dexamethasone > 7.5 mg per day). As for the types of corticosteroids, prednisone, prednisolone, and methylprednisolone are all intermediate-acting corticosteroids and are classified in one category, while dexamethasone, as a long-acting corticosteroid, is classified in another category.

### Statistical analysis

Continuous variables were displayed as mean ± standard deviation if they conformed to normal distribution; continuous variables were expressed as median within the interquartile range (IQR) if they did not conform to normal distribution. The Mann–Whitney test was used for continuous variables. Categorical variables were reported as frequencies and percentages. The Fisher exact test or chi-square tests were conducted to compare binary and categorical variables.

To compare outcomes between two groups of patients who had similar baseline characteristics except for different treatment variables (usual dose and higher dose of corticosteroids). We performed a two-to-one propensity score (PS) matching analysis estimated by logistic regression to account for potential confounding factors. Variables involved in the PS estimation included sex, age, underlying diseases, Immunocompromised condition, respiratory support, vasopressor use, and duration of corticosteroid treatment. Matching was based on the logit of the PS using nearest-neighbor matching (greedy-type matching) with a caliper width of 0.25. After matching, Kaplan–Meier curves were used to track the 28-day mortality for patients receiving the usual or higher dose of corticosteroid therapy. The effect of different doses of corticosteroids on the primary clinical outcome and other outcome events was also explored by univariate logistic regression models. In addition, an extended Cox regression model that treated corticosteroids as a time-varying exposure variable was used to assess the effect of usual or higher doses of corticosteroid treatment on 28-day mortality. The effect of different doses of corticosteroid treatment on 28-day mortality was also performed using Cox regression in the following subgroups: age < 75 years or age ≥ 75 years; different respiratory support status [no oxygen or simple oxygen, high flow nasal cannula (HFNC) or noninvasive positive pressure ventilation (NIPPV), and invasive positive pressure ventilation (IPPV) or ECMO]. For multivariate models, different doses and types of corticosteroid treatment were included for analysis.

*P* values < 0.05 were considered statistically significant. All the analyses were performed with the statistical software packages R 4.0.4 (http://www.R-project.org, The R Foundation), Statsape software (version BS2.0; Hangzhou, China), and SPSS statistics software (version 25.0; SPSS Inc., Chicago, IL).

## Results

**Fig. 1:**
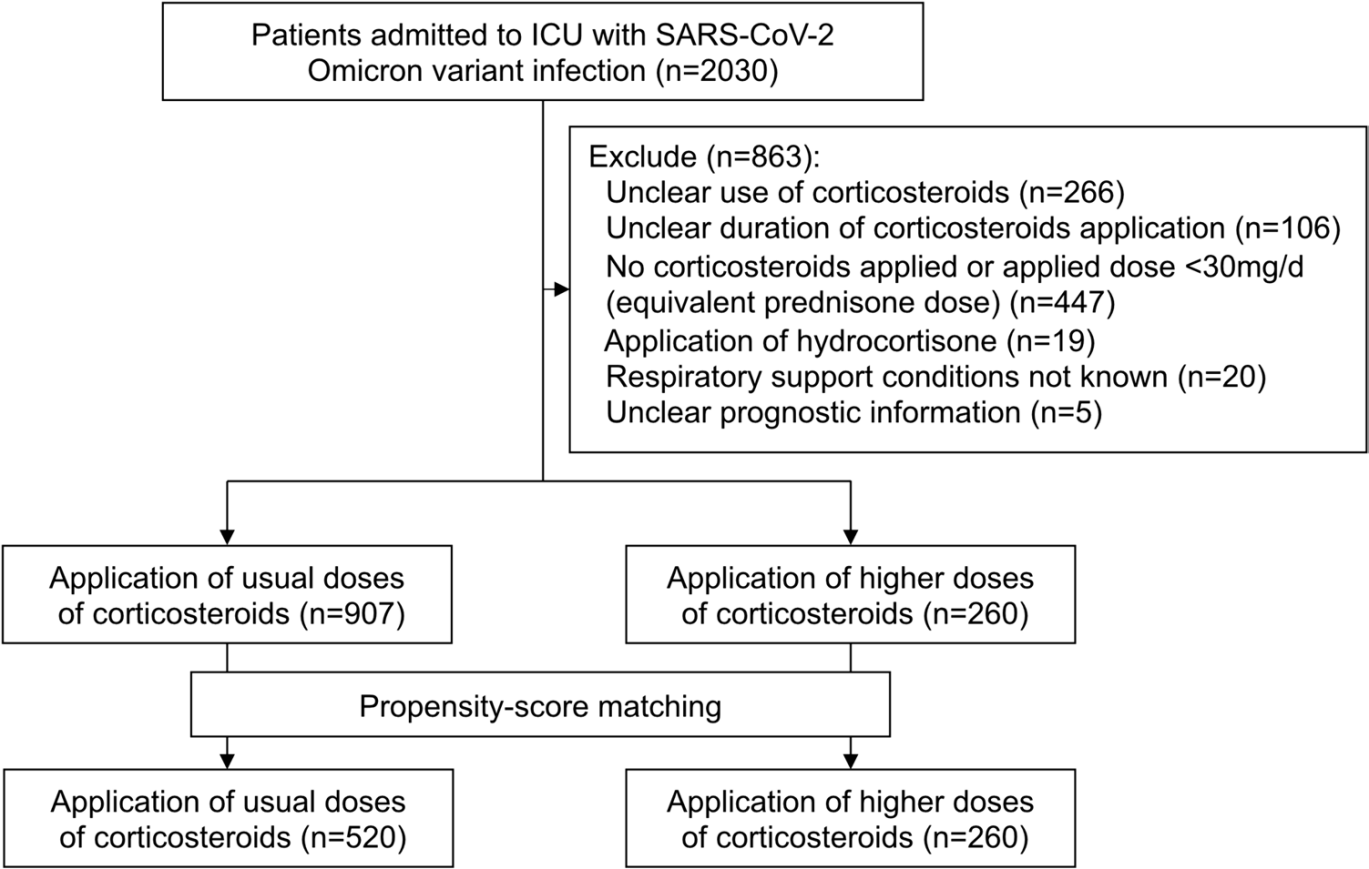
Schematic diagram of patient screening for inclusion and analysis

Between November 1, 2022, and February 11, 2023, 2030 patients diagnosed with severe or critical SARS-CoV-2 Omicron variant infection from 59 ICUs in the mainland of China were enrolled in the study. Twenty patients with missing information on respiratory support conditions and five with unclear prognostic information were excluded. The aim of this study was to determine the prognostic impact of different doses of corticosteroids, 372 patients with vague corticosteroid use (*n* = 266) and duration of use (*n* = 106) were excluded. Four hundred and forty-seven patients who had not applied corticosteroids (applied before ICU or with contraindications) or had tapered the dose to less than the usual dose were also excluded. Nineteen patients treated with hydrocortisone were excluded from the analysis due to small numbers. The detailed data-cleaning process is shown in Fig. 1.

### Clinical characteristics

**Fig.2:**
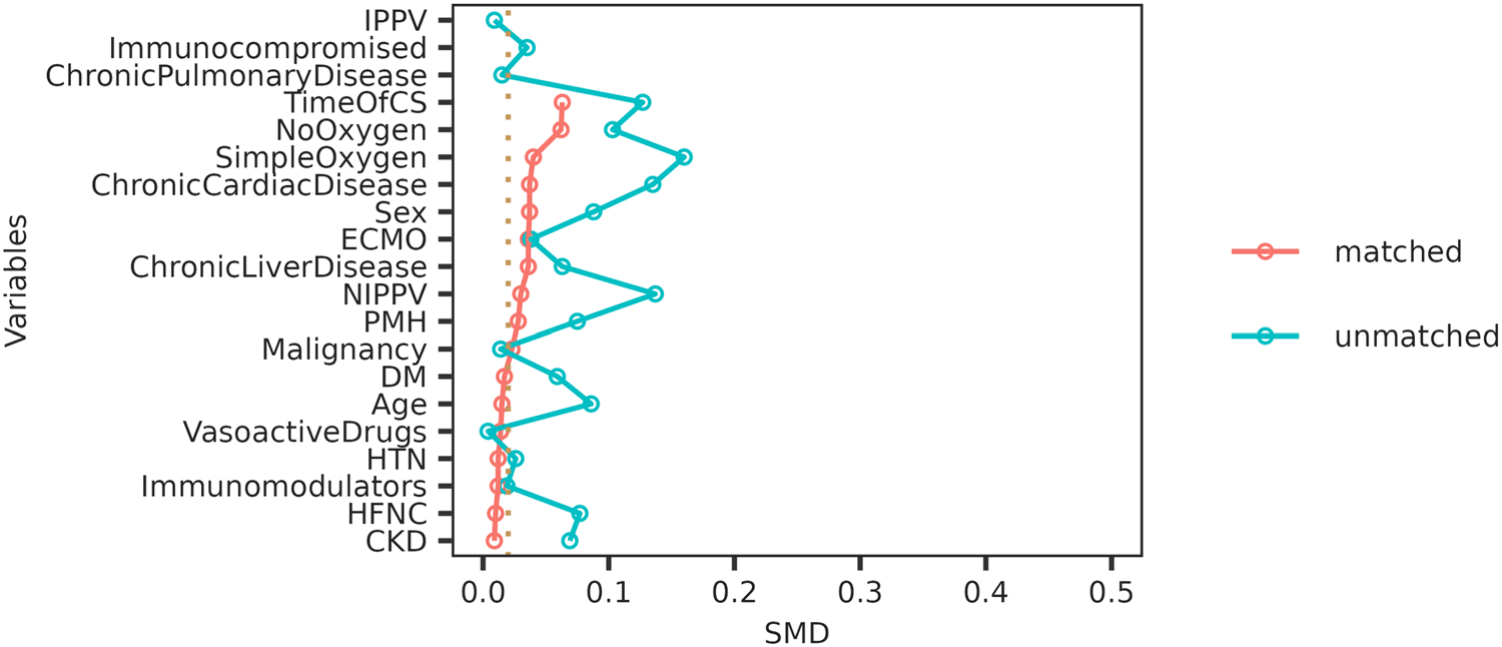
Summaries of the balance of variables before and after propensity score matching. *CKD* chronic kidney disease, *CS* corticosteroid, *DM* diabetes, *ECMO* extracorporeal membrane oxygenation, *HFNC* high flow nasal cannula; *HTN* hypertension, *IPPV* invasive positive pressure ventilation, *NIPPV* non-invasive positive pressure ventilation, *SMD* standard mean difference

Ultimately, 1167 patients with severe or critical SARS-CoV-2 Omicron variant infection were included in the final analysis, with a median age of 75 (IQR 61–82) years and 74.0% (863/1167) male, of whom 907 patients were on usual doses of corticosteroids and 260 patients on higher doses. After the propensity score matching, 520 patients on usual doses of corticosteroids and 260 patients on higher doses were matched (Fig. 1). The balanced data of variables included in the matching model are shown in Fig. 2.

In the cohort after matching, the median age was 74(IQR 66–81) years, 77.9% (608/780) were male, 91.8% (716/780) had comorbid underlying diseases, and 13.1% (102/780) were in the immunocompromised condition. The median time between patients’ symptoms onset and ICU admission was 9 (IQR 5–14) days. Within the first 24 h of admission to the ICU, 1.7% (13/780) of patients did not require oxygen, 18.2% (142/780) required simple oxygen, 19.4% (151/780) required HFNC, 27.4% (214/780) required NIPPV, 33.1% (258/780) required IPPV, and 0.3% (2/780) of patients required ECMO support. These patients had a median PaO_2_ of 66.7 (53.1–86.4) mmHg, a median PFR of 109.5 (73.2–179.6) mmHg, and median APACHE II scores and SOFA scores of 15 (11–22) and 5 (3–8), respectively. Prior to ICU admission, corticosteroids and antiviral therapy were applied in 33.8% (264/780) and 23.7% (185/780) of patients, respectively. The median duration of application was 5 (2–8) days in matched patients on corticosteroids, of whom 26.7% (208/780) were on dexamethasone, and 56.5% (441/780) and 12.4% (97/780) were combined with antiviral therapy and other immunomodulators (baricitinib and/or tocilizumab), respectively. There were no significant differences in any of these variables between patients in the usual dose corticosteroid treatment group and patients in the higher dose treatment group, suggesting that the two groups were matched in clinical characteristics. For more detailed data, see Table 1.

**Table 1:** Clinical characteristics of enrolled patients before and after propensity score matching

Features	*N*	Unmatched patients (*n* = 1167)			*N*	Propensity-score matched patients (*n* = 780)		
		Usual dose (*n* = 907)	Higher dose (*n* = 260)	*P* value		Usual dose (*n* = 520)	Higher dose (*n* = 260)	*P* value
Gender, male (*n*, %)	1167	663 (73.1)	200 (76.9)	0.215	780	408 (78.5)	200 (76.9)	0.625
Age, yrs(Median, IQR)	1167	75 (67–82)	73 (67–81)	0.148	780	74 (65–81)	73 (67–81)	0.867
Underlying disease	1167	818 (90.2)	240 (92.3)	0.300	780	476 (91.5)	240 (92.3)	0.712
Hypertension	1167	487 (53.7)	143 (55.0)	0.709	780	283 (54.4)	143 (55.0)	0.879
Chronic cardiac disease	1167	234 (25.8)	83 (31.9)	0.050	780	157 (30.2)	83 (31.9)	0.622
Chronic pulmonary disease	1167	121 (13.3)	36 (13.8)	0.833	780	72 (13.8)	36 (13.8)	1.000
Diabetes	1167	265 (29.2)	83 (31.9)	0.400	780	162 (31.2)	83 (31.9)	0.827
Chronic liver disease	1167	8 (0.9)	1 (0.4)	0.419	780	1 (0.2)	1 (0.4)	0.617
CKD	1167	64 (7.1)	14 (5.4)	0.341	780	27 (5.2)	14 (5.4)	0.910
Malignancy	1167	66 (7.3)	18 (6.9)	0.846	780	33 (6.3)	18 (6.9)	0.759
Immunocompromised host, (*n*, %)	1167	108 (11.9)	34 (13.1)	0.611	780	68 (13.1)	34 (13.1)	1.000
Time since symptom onset, days(Median, IQR)	1167	9 (5–14)	9 (5–14)	0.879	780	9 (5–14)	9 (5–15)	0.982
Respiratory support, (*n*, %)	1167				780	780		
No oxygen	1167	23 (2.5)	3 (1.2)	0.183	780	10 (1.9)	3 (1.2)	0.429
Simple oxygen	1167	235 (25.9)	50 (19.2)	0.027	780	92 (17.7)	50 (19.2)	0.600
HFNC	1167	151 (16.6)	51 (19.6)	0.265	780	100 (19.2)	51 (19.6)	0.898
NIPPV	1167	188 (20.7)	69 (26.5)	0.046	780	145 (27.9)	69 (26.5)	0.691
IPPV	1167	304 (33.5)	86 (33.1)	0.894	780	172 (33.1)	86 (33.1)	1.000
ECMO	1167	6 (0.7)	1 (0.4)	0.610	780	1 (0.2)	1 (0.4)	0.617
Vasopressor use, (*n*, %)	1167	183 (20.2)	52 (20.0)	0.950	780	107 (20.6)	52 (20.0)	0.850
PaO_2_, mmHg(Median, IQR)	1060	68.0 (54.2–88.3)	65.6 (53.0–87.1)	0.419	697	67.3 (53.4–85.0)	65.6 (53.0–87.1)	0.859
PFR, mmHg(Median, IQR)	887	120.5 (73.7–205.0)	104.5 (70.0–165.9)	0.054	598	112.1 (75.5–185.8)	104.5 (70.0–165.9)	0.238
APACHE II score, (Median, IQR)	1008	15 (11–23)	14 (11–21)	0.838	677	15 (11–22)	14 (11–21)	0.905
SOFA score, (Median, IQR)	1008	5 (3–8)	5 (3–8)	0.278	675	5 (3–8)	5 (3–8)	0.452
Corticosteroids before ICU admission, (*n*, %)	990	295 (38.4)	87 (39.4)	0.787	656	177 (40.7)	87 (39.4)	0.744
Antiviral before ICU admission, (*n*, %)	970	215 (28.6)	63 (28.8)	0.968	647	122 (28.5)	63 (28.8)	0.944
Duration of corticosteroid treatment, days(Median, IQR)	1167	4 (2–7)	5 (3–8)	0.008	780	5 (2–8)	5 (3–8)	0.175
Type of corticosteroid, dexamethasone, (*n*, %)	1167	234 (25.8)	75 (28.8)	0.326	780	133 (25.6)	75 (28.8)	0.330
Combined with antiviral therapy, (*n*, %)	1167	506 (55.8)	144 (55.4)	0.908	780	297 (57.1)	144 (55.4)	0.646
Combined with baricitinib or tocilizumab, (*n*, %)	1167	121 (13.3)	33 (12.7)	0.785	780	64 (12.3)	33 (12.7)	0.878

PaO_2_, PFR, APACHE II score, and SOFA score were based on the worst variables recorded during the first 24 h of ICU admission

*CKD* chronic kidney disease, *HFNC* high flow nasal cannula, *NIPPV* noninvasive positive pressure ventilation, *IPPV* invasive positive pressure ventilation, *ECMO* Extracorporeal membrane oxygenation, PFR PaO_2_/FiO_2_ ratio, *APACHE II score* acute physiology and chronic health evaluation II score, *SOFA score* sequential organ failure assessment score

### Mortality and other clinical outcomes analysis

**Fig. 3:**
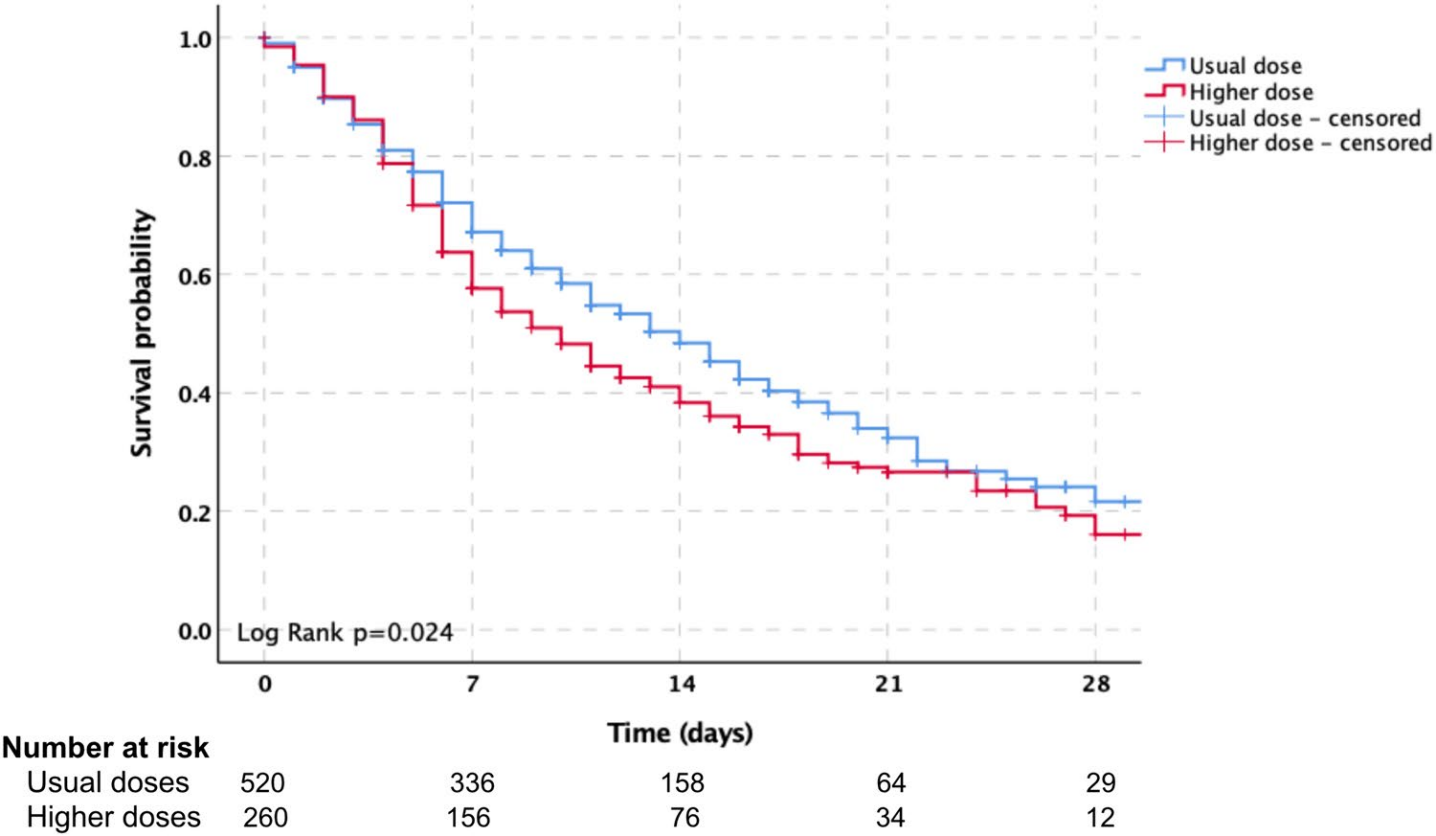
Kaplan–Meier curves of 28-day mortality for patients in the usual and higher dose corticosteroid treatment groups

After the propensity score matching, we performed logistic regression to determine the impact of higher doses of corticosteroids in patients with severe or critical SARS-CoV-2 Omicron variant infection in the ICU. The median ICU stay was 9 (5–15) and 8 (5–15) days for patients receiving usual doses and higher doses of corticosteroids, respectively. The 28-day all-cause mortality in ICU for all patients was 59.7% (466/780), with 56.0% (291/520) in the usual dose corticosteroid treatment group and 67.3% (175/260) in the higher dose corticosteroid treatment group. Kaplan–Meier curves for 28-day all-cause mortality demonstrated lower survival in patients in the higher dose corticosteroid treatment group (Log Rank *p* = 0.024) (Fig. 3). Logistic regression showed that higher doses of corticosteroid application were associated with 28-day mortality (OR = 1.62,95% CI 1.19–2.21, *p* = 0.002). The all-cause in ICU mortality for all patients was 61.4% (479/780), with 57.5% (299/520) in the usual dose corticosteroid treatment group and 69.2% (180/260) in the higher dose corticosteroid treatment group. Logistic regression also showed that higher doses of corticosteroid application were associated with in-ICU mortality (OR = 1.66,95% CI 1.21–2.28, *p* = 0.002). Other outcome events including organ dysfunction in ICU stay, secondary infection and septic shock, acute kidney injury, acute myocardial injury, deep vein thrombosis and acute pulmonary embolism, acute liver injury, gastrointestinal bleeding, hyperglycemia, and the application of renal replacement therapy and ECMO support in ICU stay were not associated with higher doses of corticosteroid therapy. The detailed data have participated in Table 2.

**Table 2:** Comparison of clinical outcomes of matched patients in usual and higher dose corticosteroid treatment groups

Outcomes	Usual dose (n = 520)	Higher dose (n = 260)	OR
Length of ICU stay, days (Median, IQR)	9 (5–15)	8 (5–15)	
28-day mortality	291 (56.0)	175 (67.3)	1.62 (1.19–2.21)
In-ICU mortality	299 (57.5)	180 (69.2)	1.66 (1.21–2.28)
Organ dysfunction in ICU stay			
Respiratory system	453 (87.1)	238 (91.5)	1.60 (0.96–2.66)
Circulation	211 (40.6)	107 (41.2)	1.02 (0.76–1.39)
Kidney	118 (22.7)	62 (23.8)	1.07 (0.75–1.52)
Liver	36 (6.9)	27 (10.4)	1.56 (0.92–2.63)
Hematologic system	61 (11.7)	38 (14.6)	1.29 (0.83–1.99)
Neurological system	27 (5.2)	12 (4.6)	0.88 (0.44–1.77)
Secondary infection	119 (22.9)	70 (26.9)	1.24 (0.88–1.75)
Septic shock	140 (26.9)	67 (25.8)	0.94 (0.67–1.32)
Acute kidney injury	242 (46.5)	109 (41.9)	0.83 (0.61–1.12)
Acute myocardial injury	89 (17.1)	51 (19.6)	1.18 (0.81–1.73)
Deep venous thrombosis	45 (8.7)	16 (6.2)	0.69 (0.38–1.25)
Acute pulmonary embolism	7 (1.3)	5 (1.9)	1.44 (0.45–4.57)
Acute liver injury	42 (8.1)	31 (11.9)	1.54 (0.94–2.52)
Gastrointestinal bleeding	35 (6.7)	12 (4.6)	0.67 (0.34–1.32)
Hyperglycemia	142 (27.3)	77 (29.6)	1.12 (0.81–1.56)
Renal replacement therapy in ICU stay	67 (12.9)	32 (12.3)	0.95 (0.61–1.49)
ECMO in ICU stay	9 (1.7)	7 (2.7)	1.57 (0.58–4.27)

ECMO: Extracorporeal membrane oxygenation

To further discuss the effect of higher doses of corticosteroids and different types of corticosteroids on 28-day mortality and time to death, we performed Cox regression analysis. The results showed that the use of higher doses of corticosteroids was still associated with 28-day mortality (HR 1.234, 95% CI 1.023–1.489, *p* = 0.028) in patients with severe or critical SARS-CoV-2 Omicron variant infection after the inclusion of time variable and was not related to the type of corticosteroid applied. Application of different types of corticosteroids (dexamethasone or methylprednisolone/prednisolone/prednisolone) had no effect on the 28-day prognosis. In subgroup analysis, no effect of higher doses and different types of corticosteroid application on 28-day mortality was observed in the subgroups of patients with age greater than 75 years old or not and different respiratory support conditions at the time of ICU admission. The detailed data are demonstrated in Table 3.

**Table 3:** Impact of corticosteroid dose and type on 28-day mortality of matched patients in usual and higher dose corticosteroid treatment groups

Variable	Univariate analysis			Multivariate analysis		
	HR	95%CI	*P* value	HR	95% CI	*P* value
All patients, *n* = 780						
Higher dose of CS	1.233	1.022–1.487	0.029	1.234	1.023–1.489	0.028
Type of CS^*^	0.898	0.732–1.104	0.307	0.897	0.730–1.101	0.298
Age < 75yrs, *n* = 402						
Higher dose of CS	1.293	0.980–1.705	0.069	1.291	0.979–1.703	0.071
Type of CS	0.803	0.578–1.114	0.189	0.804	0.579–1.116	0.192
Age ≥ 75yrs, *n* = 378						
Higher dose of CS	1.233	0.954–1.592	0.109	1.236	0.957–1.597	0.105
Type of CS	0.914	0.700–1.192	0.506	0.908	0.696–1.185	0.478
No Oxygen or Simple Oxygen, *n* = 155						
Higher dose of CS	1.321	0.770–2.268	0.312	1.323	0.770–2.273	0.310
Type of CS	0.979	0.537–1.784	0.944	0.964	0.528–1.761	0.905
HFNC or NIPPV, *n* = 365						
Higher dose of CS	1.269	0.954–1.688	0.102	1.268	0.953–1.686	0.103
Type of CS	0.856	0.621–1.179	0.340	0.857	0.622–1.180	0.344
IPPV or ECMO, *n* = 260						
Higher dose of CS	1.289	0.969–1.713	0.081	1.292	0.971–1.717	0.078
Type of CS	0.905	0.670–1.223	0.517	0.900	0.666–1.217	0.494

^*^Prednisone/prednisolone/methylprednisolone as reference in type of CS analysis

*CS* corticosteroid, *HFNC* high flow nasal cannula, *NIPPV* noninvasive positive pressure ventilation, *IPPV* invasive positive pressure ventilation, *ECMO* Extracorporeal membrane oxygenation

## Discussion

This study aimed to investigate the prognostic impact of varying corticosteroid doses on patients with severe or critical SARS-CoV-2 Omicron variant infection. The propensity score matching analysis demonstrated that higher doses of corticosteroids were associated with increased 28-day mortality in this group of patients, irrespective of the type of corticosteroid used.

The publication of the RECOVERY trial established the cornerstone role of corticosteroids (dexamethasone 6 mg daily for no more than ten days) in the treatment of COVID-19, especially in critically ill patients requiring higher respiratory support conditions (Horby et al. 2021). Several subsequent studies have clarified that corticosteroids can improve the prognosis of patients with severe or critical COVID-19 to a certain degree (Sterne et al. 2020). Based on the results of these clinical trials, the World Health Organization and National Institutes of Health guidelines for the treatment of COVID-19 strongly recommend the use of corticosteroids in patients with critically ill COVID-19 (Health 2023; Organization 2023). However, the doses of corticosteroids applied in different trials are not uniform, e.g., dexamethasone 6 mg daily and 20 mg daily may both improve the prognosis, which creates confusion in clinical practice because higher doses of corticosteroids may bring more management challenges such as secondary infections, hyperglycemia, and gastrointestinal bleeding, especially in critically ill patients admitted to the ICU (Horby et al. 2021; Tomazini et al. 2020). Therefore, several subsequent studies have explored whether higher than 6 mg of dexamethasone or equivalent doses of other types of corticosteroids can provide a prognostic benefit compared to usual doses. The COVID STEROID 2 trial, an international parallel randomized controlled trial, showed that 12 mg of dexamethasone daily did not significantly improve the 28-day life support-free rate and 180-day mortality in patients with severe COVID-19 compared to 6 mg of dexamethasone daily, but the results favored higher doses of dexamethasone (Granholm et al. 2022; Munch et al. 2021). However, the newly published RECOVERY trial showed that higher doses of dexamethasone (20 mg dexamethasone daily for 5 days, then tapered to 10 mg dexamethasone daily for 5 days for a total of no more than 10 days) resulted in an increased risk of death in patients with COVID-19 who were hypoxic but did not require mechanical ventilation compared to usual doses of dexamethasone (6 mg dexamethasone daily for no more than 10 days) (Group 2023). Another randomized double-blind controlled trial in patients with severe COVID-19 treated with methylprednisolone pulse therapy (1 g applied for 3 days) on top of usual dexamethasone therapy (6 mg dexamethasone daily for no more than 10 days) showed no prognostic benefit from higher doses of corticosteroids (Salvarani et al. 2022). Other relatively small studies also do not provide an answer to the question of whether higher doses of corticosteroids provide a prognostic benefit for patients with severe COVID-19, but the limited data suggested that higher doses of corticosteroids may be harmful (Katz et al. 2023; Toroghi et al. 2022; Yaqoob et al. 2022). Our study explored whether higher doses of corticosteroids (greater than 50 mg equivalent prednisone dose per day), using 30 to 50 mg equivalent prednisone per day as the usual doses, resulted in a prognostic benefit in patients with severe or critical COVID-19 admitted to the ICU. The results showed a higher 28-day all-cause mortality in this group of patients. This result is not contradictory to previous studies and further indicates that higher doses of corticosteroids in patients with severe or critical COVID-19 do not lead to improved prognosis.

With the continuous mutation of the SARS-CoV-2 virus, the Omicron variant is gradually replacing other variant strains as the mainstream strain of the current epidemic. Several large-scale studies have suggested that the Omicron variant caused less severe disease than the Delta variant in adults admitted to the hospital with SARS-CoV-2 infection but still resulted in high mortality (Bouzid et al. 2022; Català et al. 2022; Iuliano et al. 2022; Lauring et al. 2022). In patients admitted to the ICU, the mortality of the Omicron variant infection was not inferior to that of the Delta variant (de Prost et al. 2022). These results demonstrated that previous immunizations and vaccines might attenuate the severity of critically ill COVID-19 caused by the SARS-CoV-2 Omicron variant and that once severe or critical disease is caused by infection with the Omicron variant, the severity is not inferior to that of infection with other variants. Our study showed that patients who were critically ill and required ICU treatment due to SARS-CoV-2 Omicron variant infection had a mortality that was not inferior to that of the Alpha and Delta variant infections, which may support the above inference to some extent (Do et al. 2023; Grasselli et al. 2020). The presence of underlying disease in 94.2% of our cohort may also account for the poor prognosis of this group of patients, and similar findings have been reported in other studies (Piralla et al. 2023).

In addition, there are very limited studies on the application of corticosteroid therapy in patients with severe or critical COVID-19 due to the SARS-CoV-2 Omicron variant. Our results suggested that the application of higher doses (greater than the equivalent prednisone dose of 50 mg/d) of corticosteroids in this group of patients may lead to a poor prognosis, indicating that the application of corticosteroids in these patients should be more conservative. The reason for this finding is unknown, and a milder systemic inflammatory response due to infection with the SARS-CoV-2 Omicron variant may be one of the plausible explanations (Bojkova et al. 2022; Du et al. 2022). Besides, the median age of patients in this study reached 75 years. The possibility that elderly patients with critically ill COVID-19 may benefit less from corticosteroid therapy may be another reason for this result (Jung et al. 2021).

Our study had limitations. Firstly, it was a retrospective study with limited ICU bed availability, making the enrolled patients not fully representative of all critically ill patients. Propensity score matching was used to achieve comparability between the groups, but some patients had missing laboratory test results. Inconsistent detection reagents and ranges for certain tests further affected the analysis. Additionally, accurately obtaining the duration and dose of corticosteroids prior to ICU admission was challenging due to the study's retrospective nature, which could impact result interpretation. Secondly, it was a nonrandomized controlled study with initial differences in clinical characteristics between the groups. Although propensity score matching was performed, unmeasured or unknown confounders may still affect the results. Furthermore, the study focused only on clinical endpoints and outcomes within the ICU, without analyzing long-term outcomes.

## Conclusions

The study concluded that in severe or critical COVID-19 patients with Omicron variant infection, higher doses of corticosteroids were associated with increased 28-day and in-ICU mortality. This association was found to be irrespective of the type of corticosteroid used. These findings suggested a need for careful consideration and management of corticosteroid dosing in treating such patients to improve clinical outcomes.

## Data Availability

The data that support the findings of this study are available from QZ. These data were used under license for the current study, so these data are not publicly available. However, the data are available from the authors upon reasonable request and with permission from corresponding authors.
